# Prognostic Factors in Advanced Non-Small-Cell Lung Cancer Patients: Patient Characteristics and Type of Chemotherapy

**DOI:** 10.4061/2011/152125

**Published:** 2010-12-05

**Authors:** Salah Abbasi, Ahmed Badheeb

**Affiliations:** Clinical Oncology Department, King Hussein Cancer Center, Queen Rania Al-Abdullah Street, Al-Jubeiha, Amman 11941, Jordan

## Abstract

Eleven prognostic factors were retrospectively analyzed in 270 newly diagnosed patients with advanced non-small-cell lung cancer including age, sex, performance status, histology, stage, smoking status, hemoglobin level, forced expiratory volume in one second (FEV1), weight loss >5% in 3 months preceding therapy, number of involved organs, and type of first-line chemotherapy. Response rate was 35.6%, and median survival was 8.2 months (95% CI, 7.8 to 8.7) for the whole group. Age ≤60 years (*P* = .016), FEV1 ≥ 2L (*P* = .03), and the use of platinum/docetaxel (*P* < .0001) were significantly associated with an improved survival. Histology did not affect outcome in the absence of targeted therapies.

## 1. Introduction

Lung cancer is the leading cause of cancer deaths among both men and women in the world. Non-small-cell lung cancer (NSCLC) represents 75–85% of all lung cancer cases. Approximately, two-thirds of NSCLC patients have advanced stage at diagnosis beyond curative resection.[1] The median survival time for patients with untreated metastatic NSCLC is only 4–5 months, with a survival rate at one year of only 10% [2]. However, a meta-analysis has demonstrated that, as compared with best supportive care, chemotherapy results in a small improvement in survival in patients with advanced NSCLC [3]. Newer chemotherapy combinations showed a response rate of 19–32% and a median survival time of 7.9 to 11.3 months [4, 5]. These numbers are also improved significantly with the use of targeted therapies although results have been variable among patients. This difference in outcome among patients with the same clinical stage of the disease suggests that advanced NSCLC is a heterogeneous disease. Additionally, some patients experience weight loss and some have a significant number of comorbidities. This wide spectrum of clinical features of patients with stage IIIB/IV NSCLC probably contributes to disparities in outcomes seen in different clinical trials. Although prognostic variables have been described for advanced stage NSCLC [6], no reports have addressed this issue in our patient population. Therefore, this study aims to determine the patient and tumor variables that are associated with improved outcome in Jordanian patients who are undergoing first-line palliative chemotherapy for stage IIIB/IV NSCLC. Also, we looked at the outcome of chemotherapy in our patients with advanced NSCLC, who in general have limited access to new targeted therapies and molecular testing.

## 2. Patients and Methods

We retrospectively reviewed the clinical records of 321 patients with a pathological diagnosis of advanced, noncurative stage IIIB or IV NSCLC, who were seen and treated at King Hussein Cancer Center (KHCC) in Jordan between 2007 and 2009. More than 80% of lung cancer patients in Jordan are treated at KHCC, which makes the sample very representative of the Jordanian population.

Tumors were classified according to the recommendations of the WHO. Tumor staging was done according to the international staging system for NSCLC 1997 [7]. All patients were staged by computed tomography scan of the chest, abdomen, and pelvis and bone scan. Positron emission tomography scan and brain imaging were used for staging in some patients upon the discretion of the treating physician if needed. 

Data collected included patients gender, age at diagnosis, Eastern Cooperative Oncology Group performance status (PS), histology, stage, smoking status, history of weight loss more than 5% in 3 months preceding diagnosis, hemoglobin level (Hb) at diagnosis (in g/dL), forced expiratory volume in one second (FEV1) at diagnosis, number of involved organs by metastasis, and type of first-line chemotherapy for treated patients.

A total of 270 patients out of the 321 were treated with first-line chemotherapy (according to treating physician discretion) and were included in the analysis for response rate, survival time, and prognostic variables. Fifty one patients were not treated either because of poor PS or patient's refusal and were not included in the analysis for survival or prognosis. Time to progression (TTP) was defined as the time from first day of chemotherapy to first documentation of progressive disease. Survival time was measured from the date of first day of chemotherapy to date of death from any cause or date of last patient contact.

This work has been approved by the institutional review board at KHCC.

## 3. Statistical Analysis

All treated (*n* = 270) patients were included in the statistical calculations. Duration of survival was assessed using the Kaplan-Meier method. Variables were studied for influence on survival in a univariate analysis by using the Log-rank test, and in a multivariate analysis using a stepwise Cox proportional hazards regression analysis. The results were considered significant at the 0.05 level. Statistical analysis was performed by using the SAS version 9.1 (SAS Institute Inc., Cary, NC).

## 4. Results

### 4.1. Patient's Characteristics

Among the 321 patients with advanced NSCLC, 273 (85%) were males and 48 (15%) were females with a male : female ratio of 5.7 : 1. Two hundreds and nine patients (65%) were above the age of 60 years at diagnosis. The most common histology was adenocarcinoma (181, 56.4%), while squamous cell carcinoma and large cell carcinoma accounted for 31% and 6.2%, respectively.

Most patients were smokers at time of diagnosis (78.2%), but only 21% of patients reported a history of significant weight loss. The majority of patients had metastasis to single organ (215, 67%), rather than two organs or more (106, 33%). The median time between first symptoms appearance and actual diagnosis of lung cancer was 4 months (range, 1–12).

### 4.2. Response and Outcome of Therapy

Characteristics of the 270 patients who received palliative chemotherapy are shown in Table 1. The majority of patients received platinum/docetaxel combination (183, 67.8%), while the rest of patients received other therapies (platinum/gemcitabine in 21.9%, other platinum-based doublets in 2.6%, and single agent docetaxel in 7.8%).

The overall response rate (ORR) (complete and partial remissions) for the whole group was 35.6%, while 30% of patients achieved stable disease, for a disease control rate of 65.6%. The ORR for platinum/docetaxel group was 38% (70/183) versus 30% for all other therapies combined (26/87), and the difference was not statistically significant (*P* = .3).

**Figure 1:**
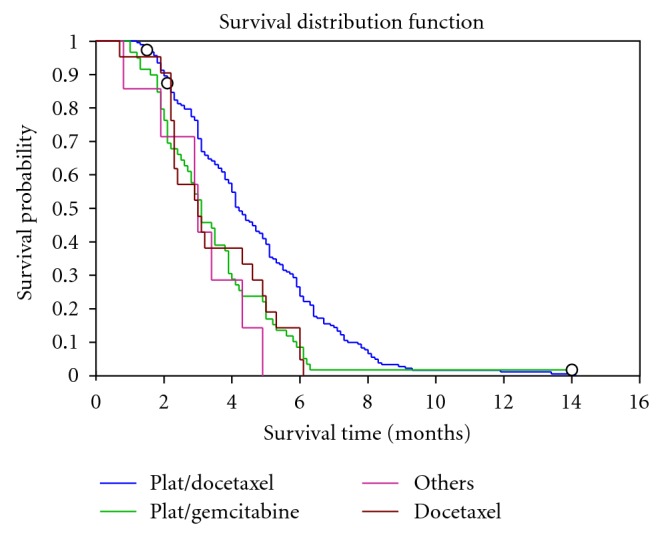
Kaplan-Meier estimate of time to disease progression among advanced NSCLC patients treated with different chemotherapy regimens.

TTP was 3.9 months (95% CI, 3.5–4.1) for the whole group of treated patients. TTP was significantly longer in the group of patients who received platinum/docetaxel (4.2 months) than for patients who received other therapies (3.1 months, *P* < .0001) as shown in Figure 1. 

**Figure 2:**
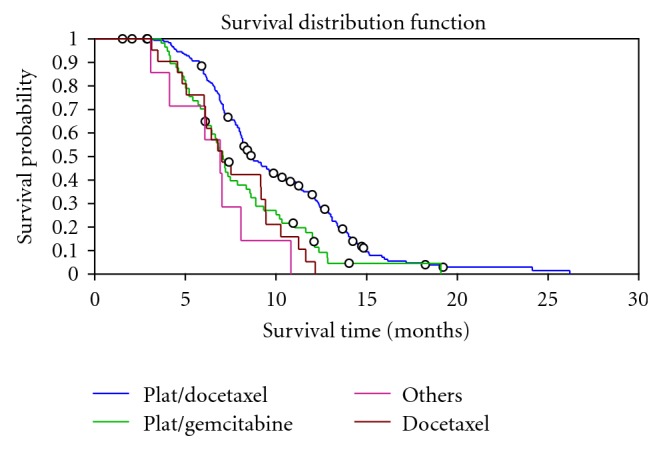
Kaplan-Meier estimate of overall survival among advanced NSCLC patients treated with different chemotherapy regimens.

The median survival time (MS) for the whole group of treated patients was 8.2 months (95% CI, 7.8–8.7) with significantly higher MS for the platinum/docetaxel group over other therapies group ( 8.9 months versus 7.1 months, respectively, *P* < .0001) as shown in Figure 2. Patients who achieved remission (partial or complete) had significantly higher MS than patients with stable disease or progressive disease (12.4 months versus 6 months, respectively, *P* < .0001).

### 4.3. Prognostic Variables

Univariate analysis of prognostic factors was performed on all treated patients with survival data (Table 1). In this analysis, the only statistically significant factors observed were age at diagnosis of 60 years or younger (*P* = .016), FEV1 at diagnosis of 2 liters or more (*P* = .03), and the use of platinum/docetaxel as first line chemotherapy (*P* < .0001). Differences in sex, PS, histology, stage, smoking status, Hb level, weight loss, or sites of metastasis were not significantly predictive of survival outcome.

The results of Cox regression analysis demonstrated the association of FEV1 ≥2L (The hazard ratio was 0.74 (95% CI, 0.55–0.92), *P* = .015), age ≤60 years (The hazard ratio was 0.61 (95% CI, 0.45–0.84), *P* = .002), and the use of platinum/docetaxel regimen (The hazard ratio was 0.48 (95% CI, 0.28–0.80), *P* = .0003) as independent prognostic factors for survival. Survival was not influenced by other prognostic variables.

## 5. Discussion

The prognosis of patients with stage IV NSCLC is still poor, most large phase III trials have shown a median survival time from 8 to 10 months [8]. In our study, we achieved an ORR of 35.6%, TTP of 3.9 months, and a median survival time of 8.2 months, consistent with international data. As expected, our outcome is lower than the median survival achieved with the use of new drugs and targeted therapies, which unfortunately are not widely available in Jordan and many other developing countries. This fact, beside the lack of molecular testing and the ability to do more costly and sophisticated genetic testing in advanced NSCLC patients, made it very important to us to look at clinical and pathological prognostic variables, which may help us in treating our patients and to better allocate our resources. 

Albain et al. demonstrated that a good PS, female sex, age ≤ 70 years, Hb level > 11 g/dL, and normal lactate dehydrogenase levels were associated with improved outcomes in patients with advanced NSCLC [9]. It is noteworthy that our study confirmed the prognostic significance of age on survival although we used a lower cutoff age (60 years, instead of 70 years) because of the lower life span of our population and earlier presentation with lung cancer (median age at presentation in this study was 62 years). One of the most important factors across many trials is PS. Patients with stage IV NSCLC who are compromised by their disease have poor survival compared to patients who are less compromised [10, 11]. Our study did not show this value for PS most probably because our patients with PS 1 or 2 did not receive treatment (small number of patients).

Current smoking during treatment for small-cell lung cancer has been shown to impact negatively on survival [12]. This present study did not find a survival advantage with respect to sex or smoking status.

Pretreatment weight loss is generally regarded as a negative prognostic factor, but not all trials have reliably shown this [13, 14]. Our study showed that weight loss more than 5% in the last 3 months preceding the diagnosis of NSCLC is not a significant prognostic variable.

FEV1 more than 2L was found to be associated with improved survival in patients with locally advanced NSCLC who undergo curative concurrent chemoradiation therapy,[6] which is consistent with our results in stage IV patients who undergo palliative chemotherapy alone.

Although our study was not designed to compare different chemotherapy regimens, it clearly showed that the use of platinum/docetaxel combination is superior to other regimens used here, and it is an independent risk factor for better TTP and MS, regardless of histology. This is especially important for our population, where new therapies like pemetrexed, bevacizumab, and other targeted therapies are costly and not readily available. Histology subtype does not reliably provide prognostic importance in patients with advanced NSCLC, especially in the absence of targeted therapies.

We hope that overall prognostic assessment and treatment decisions can be individualized by taking into consideration specific patient characteristics that have been shown to affect survival. Additionally, these prognostic factors can be used in future study designs to properly stratify patients.

## 6. Conclusion

Although our study is retrospective, includes small number of patients from single institution, and does not include molecular or genetic prognostic factors, it suggests that FEV1 ≥2L, age ≤60, and the use of platinum/docetaxel as first-line chemotherapy in stage IIIB/IV NSCLC can be useful in predicting for more favorable outcome. Histology of NSCLC does not affect outcome in the absence of targeted therapies. Future studies should look at more specific molecular and genetic factors. One study in Jordan is ongoing now to look at the prevalence of epidermal growth factor receptor mutations rate and its association with clinical response and survival in response to Erlotinib in the Jordanian population.

## Figures and Tables

**Table 1:** Characteristics and univariate analysis of prognostic factors in treated NSCLC patients (*n* = 270).

Characteristic	*N * (total = 270)	Median survival (months)	Log-rank test *P*-value
Age (years)			
≤60	108	8.6	.016
<60	162	8.0	

Sex			
Male	228	8.2	.59
Female	42	8.3	

PS			
0	243	8.3	.25
1 + 2	27	6.7	

Histology			
Adenocarcinoma	152	8.4	.31
Others	118	8.1	

Stage			
IIIB	17	11.2	.075
IV	253	8.1	

Smoking status			
Current	209	8.1	.79
Never/former	61	8.59	

Hb			
≥12	219	8.1	.41
<12	51	10.2	

FEV1			
≥2L	128	9.2	.03
< 2L	113	8.0	

Weight loss			
No	190	8.2	.89
Yes	49	8.3	

Sites of metastasis			
Single organ	191	8.7	.68
Two or more	79	8.2	

Type of chemotherapy			
Platinum/Docetaxel	183	8.9	<.0001
Platinum based doublet	66	7.1	
Docetaxel monotherapy	21	7.0	

Abbreviations: PS: performance status; Hb: hemoglobin level in g/dL; FEV1: forced expiratory volume in one second.
